# Are political views related to smoking and support for tobacco control policies? A survey across 28 European countries

**DOI:** 10.1186/s12971-017-0151-x

**Published:** 2017-12-08

**Authors:** Filippos T. Filippidis, Charis Girvalaki, Enkeleint-Aggelos Mechili, Constantine I. Vardavas

**Affiliations:** 1 Department of Primary Care and Public Health, School of Public Health, Imperial College, 310 Reynolds Building, St. Dunstan’s Road, W6 8RP, London, UK; 2 Center for Health Services Research, School of Medicine, National and Kapodistrian University of Athens, Athens, Greece; 3 Laboratory of Toxicology, Medical School, University of Crete, Rethimno, Greece; 4 Department of Healthcare, Faculty of Public Health, University of Vlora, Vlora, Albania; 5 Institute of Public Health, American College of Greece, Athens, Greece

**Keywords:** Smoking, Europe, Politics

## Abstract

**Background:**

General political views are rarely considered when discussing public support for tobacco control policies and tobacco use. The aim of this study was to explore potential associations between political views, smoking and support for tobacco control policies.

**Methods:**

We analysed responses from 22,313 individuals aged ≥15 years from 28 European Union (EU) member states, who self-reported their political views (far-left [1–2 on a scale 1–10]; centre-left (3-4); centre (5-6); centre-right (7-8); and far-right (9-10) in wave 82.4 of the Eurobarometer survey in 2014. We ran multi-level logistic regression models to explore associations between political views and smoking, as well as support for tobacco control policies, adjusting for socio-demographic factors.

**Results:**

Compared to those placing themselves at the political centre, people with far-left political views were more likely to be current smokers (Odds Ratio [OR] = 1.13; 95% Confidence Interval [CI]: 1.01–1.26), while those in the centre-right were the least likely to smoke (OR = 0.84; 95% CI: 0.76–0.93). Similar associations were found for having ever been a smoker. Respondents on the left side of the political spectrum were more likely to support tobacco control policies and those on the centre-right were less likely to support them, as compared to those at the political centre, after controlling for smoking status.

**Conclusions:**

General political views may be associated not only with support for tobacco control policies, but even with smoking behaviours, which should be taken into account when discussing these issues at a population level. Further research is needed to explore the implications of these findings.

## Background

Each year, more than 700,000 Europeans die from tobacco-related illnesses making tobacco use the leading cause of premature death and the largest threat to public health in Europe [1]. Smoke-free legislation and tax increases on tobacco have been effective in reducing smoking prevalence, mortality and healthcare expenses in many European Union (EU) member states (MS) [2]; however, the extent of tobacco control measures and smoking prevalence vary widely among EU MS [3, 4]. Socioeconomic and demographic factors associated with smoking behaviour and the attitudes towards tobacco control policies in Europe are well documented [4].

So far, little attention has been given in political epidemiology which is an emerging field which aims to depict the role of politics in shaping health outcomes of citizens [5] mainly through the policies that governments adopt [6]. Research has been performed in terms of political views of governments and the health control policies they implement [7]. It has also been shown that conservative individuals/areas have better health, an outcome which may be a result of the higher wealth of these individuals or areas [8, 9]. Some of this evidence comes from European data, where lower income individuals are more likely to vote left and higher income individuals to vote right [10]. On the contrary in the US, rich states tend to vote democratic and poor states tend to vote republican [11]. In addition, studies on the impact of political ideology on health have shown that on average health tends to be better in areas where the majority of the population is conservative oriented [12, 13].

It has been hypothesized that health behaviours may be influenced by the political ideology of individuals [9]. For example, in the US, republicans are less likely to be smokers (OR 0.85) compared with democrats [8]. Similarly, individuals who live in more conservative ideologically states are more resistant to behaviour change regardless of price increases and restrictions on smoking and more often show defiance of these policies compared with individuals who live in more liberal states [14], while citizen ideology has also been associated with enacting statewide clean indoor air laws in the United States [15].

This above evidence suggests that political ideology may be associated with the implementation of tobacco control policies and health behaviours, including smoking, in multiple levels. However, studies with individual level data are scarce and little research has been done at a European level. The aim of this study was to explore potential associations between political views, smoking and support for tobacco control policies across the 28 EU EU MS countries.

## Methods

### Data source

We analysed data from the Eurobarometer survey, collected through personal interviews in all 28 EU member states. Eurobarometer is a survey conducted by the European Commission multiple times every year covering political, social and health topics. Wave 82.4 of the survey, which was conducted in November–December 2014 included questions on tobacco use and political views [16]. A total of 27,801 individuals aged ≥15 years were interviewed, but only those who provided information regarding their political views were analysed (*n* = 22,313). Eurobarometer follows a multi-stage random sampling and the sample is representative of the EU population aged 15 years or older with regard to age, sex and area of residence. At the first stage, primary sampling units (PSU) were selected from each region within each member state, proportional to population size. The second stage included the random selection of starting addresses in each PSU, and finally households were systematically selected following a standard random route. Post-stratification and population size weighting were applied in each member state using official Eurostat data on age, sex and area of residence.

### Measures

All participants were asked “Regarding smoking cigarettes, cigars or a pipe, which of the following applies to you?”. Individuals who chose the response “You currently smoke” were classified as current smokers, those who selected the response “You used to smoke but you have stopped” were classified as former smokers and those who responded that “they have never smoked” were classified as never smokers. Current and former smokers were jointly considered ever smokers.

Participants were asked if they would be in favour or opposed (or don’t know) to each of the following tobacco control policies: banning advertising of tobacco products in shops or points of sales; increasing taxes on tobacco products; banning colours, logos and promotional elements from tobacco products packaging; banning flavours that make tobacco products more attractive; keeping tobacco products out of sight in shops or points of sale; improving the traceability of tobacco products in order to reduce their illicit trade even if this makes them a few cents more expensive (reducing illicit trade); banning the sales of tobacco via the Internet; banning the use of electronic cigarettes in environments where smoking is prohibited. For our analyses, those opposed and those who responded “don’t know” were classified as “not in favour”.

Political views were assessed with the question “In political matters people talk of "the left” and “the right". How would you place your views on this scale?”. Respondents were asked to place their views on a scale ranging from 1 (left) to 10 (right). Based on their responses, we classified participants’ political views as far-left (1–2 on the scale); centre-left (3–4); centre (5–6); centre-right (7–8); and far-right (9–10).

Self-reported data on the participants’ age (18–24; 25–39; 40–54; or ≥55 years); sex (female; or male); area of residence (urban; or rural); difficulty to pay bills (never/almost never; or from time to time/most of the time); age at which they stopped full-time education (≤15; 16–19; or ≥20 years); marital status (married/single living with partner; unmarried; or divorced/separated/widowed); self-reported social class (working class; lower middle class; middle class; or upper middle/higher class), and occupation (manual workers; other non-manual workers; self-employed; and managers) were also collected in the survey.

### Statistical analysis

Separate multi-level regression models (country being the higher level of analysis) were fitted to assess the association of political views (independent variable) with being a current and ever smoker respectively (dependent variables). Both models were adjusted for all socio-demographic variables described above. Similar multi-level logistic regression models were fitted with support for each tobacco control policy assessed being a dependent variable. The latter models were further adjusted for smoking status. Weights provided in the official dataset were considered for all descriptive analyses to reflect the complex sampling design of the survey. Logistic regression results are presented as adjusted Odds Ratios (aOR) with 95% Confidence Intervals (CI). All analyses were conducted with STATA 14.0.

## Results

Among the sample of 22,313 participants in 2014, 26.2% were current smokers and 47.1% were ever smokers. With regard to political views, 10.0% placed themselves in the far left, 24.0% in centre-left, 43.7% in the centre, 15.8% in centre-right and 6.5% in the far-right.

Table 1 describes the association between the political views of the respondents and smoking. Compared to those placing themselves at the political centre, people with far-left political views were more likely to be current smokers (OR = 1.13; 95% 95% CI: 1.01–1.26), while those in the centre-right were the least likely to smoke (OR = 0.84; 95% CI: 0.76–0.93). Similar associations were found for having ever been a smoker, where respondents with far-left political beliefs where more likely to ever have been smokers (OR = 1.20; 95% CI: 1.09–1.32) and those with centre-right beliefs were less likely to ever have been smokers (OR = 0.88; 95% CI: 0.81–0.95) compared with those placing themselves in the political centre.

**Table 1:** Association between political views and smoking across the 28 European Union Member States in 2014 (*n* = 22,313)

	Current smoker		Ever smoker	
	%	OR (95% CI)	%	OR (95% CI)
Centre (reference)	27.5	–	46.9	–
Far left	28.8	*1.13 (1.01–1.26)*	53.6	*1.20 (1.09–1.32)*
Centre left	23.8	0.93 (0.85–1.02)	46.4	1.02 (0.95–1.11)
Centre right	23.1	*0.84 (0.76–0.93)*	43.9	*0.88 (0.81–0.95)*
Far right	30.0	1.11 (0.98–1.26)	47.8	1.05 (0.94–1.17)

Multi-level logistic regression adjusted for age, sex, education, area of residence, financial status, occupation, marital status and social class

Confidence intervals in italics indicate statistically significant results

Notably people on the left side of the political spectrum were more likely and those on the centre-right less likely to support tobacco control policies, as compared to those at the political centre and after controlling for smoking status and socio-demographic factors (Table 2). Specifically, for advertising bans, those at the centre-left were more likely to support them (OR = 1.10; 95% CI: 1.01–1.19) while those from centre-right (OR = 0.87; 95% CI: 0.79–0.94) and far-right (OR = 0.85; 95% CI: 0.75–0.95) were less likely, compared with those from the political centre. Individuals placing themselves in centre-right were also less likely to support flavour bans (OR = 0.86; 95% CI: 0.79–0.94) and plain packaging policies (OR = 0.90; 95% CI: 0.83–0.98) compared with individuals at the political centre. Compared to those placing themselves at the political centre, individuals with centre-left political orientation were more likely to support placing tobacco out of sight (OR = 1.11; 95% CI: 1.02–1.20) on the contrary to individuals from centre-left (OR = 0.88; 95% CI: 0.81–0.96) and far-right (OR = 0.88; 95% CI: 0.78–0.98). Finally, centre-right oriented people were less likely to support the ban of online sales of tobacco products (OR = 0.89; 95% CI: 0.81–0.96).

**Table 2:** Association between political views and public support for tobacco control policies across the 28 European Union Member States in 2014 (*n* = 22,313)

Political views	Advertising bans	Ban flavours	Plain packaging	Raise tobacco taxes	Tobacco out of sight	Ban online sales	Ban e-cigarettes in public places
	OR (95% CI)	OR (95% CI)	OR (95% CI)	OR (95% CI)	OR (95% CI)	OR (95% CI)	OR (95% CI)
Centre (ref.)	–	–	–	–	–	–	–
Far left	1.06 (0.96–1.18)	1.06 (0.96–1.17)	1.07 (0.97–1.19)	0.99 (0.89–1.10)	0.96 (0.87–1.06)	1.03 (0.93–1.14)	1.00 (0.90–1.10)
Centre left	*1.10 (1.01–1.19)*	1.02 (0.94–1.10)	1.01 (0.94–1.10)	1.06 (0.97–1.15)	*1.11 (1.02–1.20)*	0.97 (0.89–1.05)	0.97 (0.90–1.05)
Centre right	*0.87 (0.79–0.94)*	*0.86 (0.79–0.94)*	*0.90 (0.83–0.98)*	0.96 (0.88–1.05)	*0.88 (0.81–0.96)*	*0.89 (0.81–0.96)*	0.97 (0.89–1.05)
Far right	*0.85 (0.75–0.95)*	0.97 (0.87–1.09)	1.01 (0.90–1.13)	0.99 (0.88–1.11)	*0.88 (0.78–0.98)*	1.04 (0.92–1.16)	1.03 (0.92–1.15)

Multi-level logistic regression adjusted for age, sex, education, area of residence, financial status, occupation, marital status, social class, and smoking

Confidence intervals in italics indicate statistically significant results

## Discussion

Our findings indicate that compared to those placing themselves at the political centre, people with far-left political views were more likely to be current or ever smokers, while people on the centre-right were less likely to support most tobacco control policies, compared to those from the political centre.

The association we found between political views and smoking adds to existing literature on the topic. A study conducted in the US found that more liberal state ideology predicts lower adult smoking prevalence [17] while Subramanian and Perkins found that republicans were less likely to be smokers [8]. This implies that there might be factors influencing smoking behaviour beyond those usually considered in public health which need to be further investigated. Political orientation of a subject might reflect personal attitudes towards substance use, health and the perceived role of policy measures. Alternatively, they might be an indicator of a certain lifestyle, the social environment of respondents and the peers with whom they interact [18]. Comparisons between Europe and the US is not straightforward, as the political landscape is considerably different; the division between democrats and republicans may not directly correspond to the left-right division in Europe. This could be particularly true in health policy issues, where the EU and the US have distinctively different approaches.

Our results also indicated that people with centre-right political background are less likely to support tobacco control policies, which may be related to the fact that people who consider themselves conservatives are more resistant to smoking behaviour change regardless of price increases and restrictions on smoking; they are also more likely to show defiance of these policies [14]. Support for tobacco control policies can be determined by factors beyond health considerations, including ethical and ideological beliefs about the role of the state, autonomy and liberty [19], which may differ among people across the political spectrum. Previous research has shown that, on average, health trends are better in areas where the majority of the population is conservative oriented [12, 13], but health may be influenced not only by public health policies, but also by the quality of healthcare and individual behaviours, which could also be impacted by political ideology.

This study is one of the few which have investigated the association of political orientation with smoking behaviour and support for tobacco control policies in Europe. Political ideology was assessed across a simplified left-to-right axis which may not reflect nuanced views regarding libertarianism and interference of the state. Moreover, the political environment in Europe is rapidly changing and self-identification as left or right might not be consistent between EU MS countries. The question used to assess smoking included cigars and pipes, users of which may differ from cigarette smokers. We could not distinguish between them, but exclusive use of cigars and pipes is rare, therefore the question must have predominantly captured cigarette smokers. The large and representative sample of the Eurobarometer survey, the European Commission’s surveillance system, allowed us to adjust for major confounding factors and allow potential generalisation to the entire EU population.

## Conclusion

We found that political views are associated with both tobacco use and support for tobacco control policies in the EU. There is a robust body of evidence that tobacco control policies are effective, but the level of implementation is not yet satisfactory in many parts of the world, including the European Union [2]. Tobacco industry interference is still prevalent [20] and frequently cited as the main reason for this, but our findings highlight the potential importance also of citizen political views in understanding and planning tobacco control measures at a population level. Further research on the topic is required especially in light of the implementation of policies included in the EU Tobacco Product Directive implemented in 2017 [21].

## Abbreviations

aOR: Adjusted Odds Ratio
CI: Confidence Interval
EU: European Union
MS: Member States
OR: Odds Ratio
PSU: Primary Sampling Units

## References

[1] European Commission Tobacco Policy. 2014. http://ec.europa.eu/health/tobacco/policy/index_en.htm. Accessed 10 Juns 2017.

[2] World Health Organization. WHO report on the global tobacco epidemic 2015: raising taxes on tobacco. 2015. http://apps.who.int/iris/bitstream/10665/178574/1/9789240694606_eng.pdf. Accessed 10 June 2017.

[3] Joossens L, Raw M. The tobacco control scale 2016 in Europe. 2016. http://www.cancer.be/sites/default/files/tobacco_control_scale.pdf. Accessed 10 June 2017.

[4] European Commission: Special Eurobarometer 429. Attitudes of Europeans towards tobacco. In.; 2015.

[5] Pega F, Kawachi I, Rasanathan K, Lundberg O (2013). Politics, policies and population health: a commentary on Mackenbach, Hu and Looman (2013). Soc Sci Med.

[6] Pabayo R, Kawachi I, Muennig P (2015). Political party affiliation, political ideology and mortality. J Epidemiol Community Health.

[7] Macinko J, Silver D (2015). Diffusion of impaired driving Laws among US states. Am J Public Health.

[8] Subramanian SV, Perkins JM (2010). Are republicans healthier than democrats?. Int J Epidemiol.

[9] Subramanian SV, Huijts T, Perkins JM (2009). Association between political ideology and health in Europe. Eur J Pub Health.

[10] Dorling D, Smith GD, Shaw M (2001). Analysis of trends in premature mortality by labour voting in the 1997 general election. BMJ.

[11] Gelman A, Shor B, Bafumi J, Park D (2008). Rich state, poor state, red state, blue state: What's the matter with Connecticut?. Quarterly Journal of Political Science.

[12] Kelleher C, Timoney A, Friel S, McKeown D (2002). Indicators of deprivation, voting patterns, and health status at area level in the Republic of Ireland. J Epidemiol Community Health.

[13] Page A, Morrell S, Taylor R (2002). Suicide and political regime in new South Wales and Australia during the 20th century. J Epidemiol Community Health.

[14] Gruber J, Mullainathan S (2002). Do cigarette taxes make smokers happier? National Bureau of economic research working paper series.

[15] Tung GJ, Vernick JS, Stuart EA, Webster DW (2014). Political factors affecting the enactment of state-level clean indoor air laws. Am J Public Health.

[16] European Commission: Eurobarometer 82.4, November–December 2014. GESIS Data Archive: ZA5933, dataset version 5.0.0 (2014). In.: TNS OPINION & SOCIAL, Brussels; 2014.

[17] Fox AM, Feng W, Yumkham R (2017). State political ideology, policies and health behaviors: the case of tobacco. Soc Sci Med.

[18] Filippidis FT, Agaku IT, Vardavas CI (2015). The association between peer, parental influence and tobacco product features and earlier age of onset of regular smoking among adults in 27 European countries. Eur J Pub Health.

[19] Anker TB (2016). Analysis of the paternalistic justification of an agenda setting public health policy: the case of tobacco plain packaging. Public Health Ethics.

[20] Fooks GJ, Smith J, Lee K, Holden C (2017). Controlling corporate influence in health policy making? An assessment of the implementation of article 5.3 of the World Health Organization framework convention on tobacco control. Glob Health.

[21] European Comission. Directive 2014/40/EU Of The European Parliament and of the Council of 3 April 2014 on the approximation of the laws, regulations and administrative provisions of the Member States concerning the manufacture, presentation and sale of tobacco and related products and repealing Directive 2001/37/EC. 2014. http://ec.europa.eu/health/tobacco/docs/dir_201440_en.pdf. Accessed 10 June 2017.

